# Trade-in Strategy and Competition between Two Independent Remanufacturers

**DOI:** 10.3390/ijerph18136745

**Published:** 2021-06-23

**Authors:** Zhangwei Feng, Na Luo, Yanping Liu

**Affiliations:** 1 School of Business, Ningbo University, Ningbo 315211, China; fengzhangwei@nbu.edu.cn; 2 Department of Information Systems and Operations Management, The University of Auckland Business School, Auckland 1142, New Zealand; n.luo@auckland.ac.nz; 3 Department of Management Science and Engineering, Business School, Nankai University, Tianjin 300071, China

**Keywords:** circular economy, remanufacturing, trade-in strategy, competition, independent remanufacturers, optimization

## Abstract

Trade-in strategy is a common mode of promotion for firms taking part in the circular economy because it encourages consumers to buy remanufactured products, via a “trade-old-for-remanufactured” framework. This paper studies trade-in strategy by developing game models for a closed-loop supply chain with one manufacturer and two independent remanufacturers. The former is responsible for producing and selling new products and the latter two compete with each other for the collection of used products and the sales of remanufactured products. Unlike the extant literature, this paper focuses on the competition between two independent remanufacturers (IRs) and studies on how holder segment (whether or not to own used products) and competition affects the trade-in strategy. It finds that the proportion of holders on the remanufactured product prices of the IR1 and IR2 have a negative correlation. Conversely, the impact of the proportion of holders on the IR1’s and IR2’s demands (and on their profits) is the opposite. The trade-in strategy generates more benefits for the IR1 only when the proportion of holders is sufficiently high. In addition, when consumers experience a greater difference in durability between remanufactured products and original new products, trade-in strategy is more welcomed by consumers, which in turn, generates more benefits for the IR1.

## 1. Introduction

With the development of remanufacturing technology, many manufacturers have started recycling and remanufacturing waste products, including end-of-life home appliances and used electronic products. According to a joint study by the United Nations University, the International Telecommunication Union, and the International Solid Waste Association, 45 million tons of waste electrical and electronic equipment (WEEE) were produced worldwide in 2016, but the recycling rate is shockingly low—only about 20%. In that year, China’s WEEE alone reached 7.2 million tons, overtaking the United States to become the world’s largest source of WEEE. Gold, silver, copper, platinum, palladium and other metals, worth some 55 billion Euros, are wasted as TVs, mobile phones and other products which are rarely recycled [1]. In 2020, about 16 million used products, including mobile phones, TVs, printers, air conditioners, refrigerators, washing machines and computers, are scrapped, generating about 800 million tons of solid waste [2]. In addition to resources being wasted, this process of scrapping products has a severe environmental impact. The development of the recycling and remanufacturing industry therefore represents significant benefits for promoting the economy, and in conserving natural resources and ecological systems.

Increasingly, remanufactured products are receiving greater attention as consumers become more conscious of the importance of sustainable practices [3]. Many manufacturers are now willing to take back used products to produce remanufactured products in a closed-loop supply chain (CLSC) or in a remanufacturing system, due to the economic and environmental benefits at hand [4,5]. For example, Hewlett–Packard (HP) proposed a global collection plan, the HP Planet Partners Program [6], to ensure 80% of original ink cartridges are collected. However, remanufactured products are generally less marketable than brand new products and consumers may be more hesitant to buy products that have already had some form of use. In order to encourage consumer uptake of remanufactured products, and to increase the collection of used products, many manufacturers now adopt trade-in strategies, including “trade-old-for-new” or “trade-old-for-remanufactured” deals [7,8,9]. The first strategy enables consumers to receive a discount on new products when they trade in used products. While the second strategy involves a discount on remanufactured products when consumers trade in used products.

To date, most studies in this area have focused on the “trade-old-for-new” strategy. In contrast, our paper investigates the selection mechanisms involved in a “trade-old-for-remanufactured” strategy as it can further promote resource conservation in both the collection of used products and the sales of remanufactured products. Our study investigates the trade-in strategy between two independent remanufacturers (IRs) and examines how this strategy is impacted by the holder segment and the competition between the IRs. To the best of our knowledge, this study is one of only a few that consider the holder segment and competition in relation to optimizing sustainable trade-in operations. To achieve this objective, we develop a CLSC with one manufacturer and two IRs, wherein the IR1 provides a strategy of trading used products for remanufactured products and the IR2 does not, in the context of both competing with each other on the sale of remanufactured products.

The remainder of the paper is organized as follows. Section 2 reviews the related literature and Section 3 introduces the basic model. Section 4 provides an equilibrium analysis and Section 5 covers some numerical experiments. Section 6 offers managerial insights and future research directions.

## 2. Literature Review

Our study is closely related to the following three research streams: Reverse logistics management, competition in remanufacturing and trade-in strategy.

### 2.1. Reverse Logistics Management

Reverse logistics is an important area for many manufacturers because it focuses on the end of the product life cycle, and thus offers insights about the changing needs of logistics with respect to the life of a product [10]. Reverse logistics, in conjunction with forward sales logistics, forms a closed-loop supply chain, in which remanufacturers and consumers can make benefits decisions for environment and economy [11,12]. Reverse logistics management can be divided into two areas: recycling and remanufacturing. Studies of recycling have largely focused on recycling channels or channels leadership. For example, Karakayali, Emir-Farinas and Akcali [13] develop a model of centralized, remanufacturer-driven, and collector-driven decentralized channels to determine the optimal acquisition price of end-of-life products. They also discuss how decentralized channels can be coordinated to reach the collection rate achieved in the centralized channel. Choi, Li and Xu [14] examine the performance of closed-loop supply chains under different channel leadership and find that the retailer-led model is the most effective. Considering some certain circumstances, e.g., competition [15], governance mechanisms [3], dual sales channel [16], they study the recycling channel selection or closed-loop supply chain leadership. Other studies have examined recycling decisions, such as the price of the end-of-life products, the collection rate, and recovery efforts [17,18]. Jian, Xu and Zhou [19] investigated the collaborative strategies in a collection system involving a third-party collector and an e-retailer, using the “Internet + recycling” business model. With respect to remanufacturing, studies have covered remanufacturing supply chain or system joint with leadership [20], two-stage remanufacturing [21], the cost construct of remanufactured products [22] and carbon tax policies [23].

### 2.2. Competition in Remanufacturing

There have been several noteworthy studies that have examined competition in remanufacturing. Majumder and Groenevelt [24], Ferguson and Toktay [25], Ferrer and Swaminathan [26], and Örsdemir et al. [27] have looked at the competition that exists between the sales of new and remanufactured products. Other studies have focused on the selection of third-party remanufacturing modes (outsourcing and authorization) and examined how the different modes affect the competition between new and remanufactured products. For example, Karakayali et al. [13] indicate that the manufacturer should pay more attention to its outsourcing decisions if environmental regulations specify target collection rates for individual quality groups. Oraiopoulos et al. [28] examine the manufacturer’s decisions and analyze the optimal licensing fee when the third-party conducts remanufacturing activities. Further, Zou et al. [29] and Feng et al. [5] cover both outsourcing and authorization modes. For the competition between different remanufactured products, Kleber et al. [30] consider two IRs which compete for the acquisition of used products and the sales of remanufactured products. One IR has a market advantage (i.e., consumers have a higher willingness-to-pay for it) and the other has an acquisition advantage (i.e., sellers prefer selling to one firm at the same acquisition price). The study found that a market advantage is significantly more powerful than an acquisition advantage.

### 2.3. Trade-in Strategy

Trade-in strategies generally constitute either a process of trading old products for new products or for remanufactured products, i.e., “trade-old-for-new” or “trade-old-for-remanufactured” strategies. Most researchers have focused on the first strategy [7,8,9,31,32], including Xiao [8], who considers the choice behavior of customers in conjunction with optimal pricing and remanufacturing decisions for firms that adopt the exchange-old-for-new program. Zhang and Zhang [9] study how the strategic purchasing behavior of consumers affects the economic and environmental values of a trade-in remanufacturing strategy. Rao et al. [31] develop an analytical model that incorporates key features of real-world durable goods markets and study a trade-in strategy amounts to an intervention by the firm in the used goods market. A handful of researchers have compared the “trade-old-for-remanufactured” strategy with the “trade-old-for-new” strategy [33,34]. Ma et al. [33], for example, consider the coexistence of these two strategies, and identify the thresholds that determine whether the firm should offer both simultaneously. The study finds that adopting two types of trade-in strategies simultaneously does not necessarily benefit the firm; rather, the firm should use different trade-in schemes under different conditions. Li et al. [34] establish a two-period model in which a monopolistic manufacturer offers a trade-in strategy to improve sales and collect used products, and to remanufacture these used products and resell them to a secondary market.

### 2.4. Uniqueness and Contributions

The literature review (summarized in Table 1) demonstrates the minimal research on trade-in strategy from the consumer perspective with respect to competition. Most studies have noted the competition between new and remanufactured products, whereas we consider the competition between remanufactured products and study the trade-in strategy involved. We do this in the context of two different situations: When there is an oversupply of used products and when all used products are collected. We seek to determine the economic benefits and behavioral characteristics involved in the trade-in strategies employed by two IRs.

## 3. The Basic Model

We consider a closed-loop supply chain (CLSC) with one manufacturer and two independent remanufacturers (IRs). The manufacturer is responsible for producing and selling new products and two IRs compete with each other for the collection of used products and the sales of remanufactured products. We do not explicitly model the manufacturer’s pricing on the new product, and instead focus solely on the competition between two IRs, to ensure a tractable model and for parsimony [30]. In our model, the manufacturer provides the quantity constraint of collecting used products. In order to model the competition between two IRs, we have the IR1 providing a trade-in strategy of trading used products for remanufactured products while the IR2 does not. Table 2 provides a summary of the parameters and variables used in our model.

This paper assumes that the scale of potential consumption is normalized to one and each consumer demands at most one unit of the remanufactured product from two IRs [30,37]. Similar to the green segment [4], we assume that there are two types of consumers (whether to own used products or not): *holders* and *non-holders*. In particular, holders, who already own a product that has been used and could be potentially traded in for a remanufactured product, and non-holders, who do not own an equivalent used product that could be potentially traded in. Therefore, non-holders can either choose to buy a remanufactured product or not buy. The proportion of holders is β, 0≤β≤1, so the proportion of non-holders is 1−β. Holders have three choices: To accept the trade-in strategy and buy the IR1’s remanufactured products, or buy the IR2’s remanufactured products, or simply retain the used products. Similar to Atasu et al. [4] and Jiang and Tian [38], a consumer with WTP θ is willing to pay for a remanufactured product from the IR1. Without loss of generality, the IR1 has a market advantage as follows, so we assume that the consumers’ WTP discount rate for remanufactured products from the IR2 is γ<1 as consumers do not view the IR2’s remanufactured products as perfect substitutes for new products [35]. In addition, we assume that the durability of new products is lower than the consumer’s WTP, i.e., 0<α<γ. As a result of these assumptions, holders obtain utilities $U_{r1}=\theta -p_{r1}+p_{u}$, $U_{r2}=\gamma \theta -p_{r2}$, and $U_{u}=\alpha \theta$, respectively [4,26,34]. Non-holders have two choices: Buy remanufactured products from the IR2 or not at all.

For holders, setting two utilities $U_{r1}$ and $U_{r2}$ to equality yields $\theta_{r12}=\frac{p_{r1}-p_{u}-p_{r2}}{1-\gamma }$ which is indifferent between two remanufactured products. Similarly, $\theta_{r1u}=\frac{p_{r1}-p_{u}}{1-\alpha }$ (or $\theta_{r2u}=\frac{p_{r2}}{\gamma -\alpha }$) which is indifferent with respect to consumers buying the IR1’s (or the IR2’s) remanufactured products and retaining used products. For non-holders, $\theta_{r2}=\frac{p_{r2}}{\gamma }$ which is indifferent with respect to consumers buying a remanufactured product from the IR2 and not buying a product at all. Given the proportion of holders β and the uniform distribution for θ, the demand for remanufactured products are as follows in Table 3.

**Figure 1:**
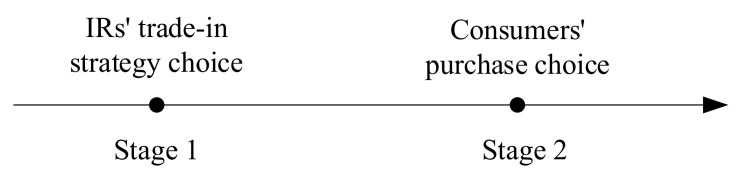
Game sequence of IRs and consumers.

**Figure 2:**
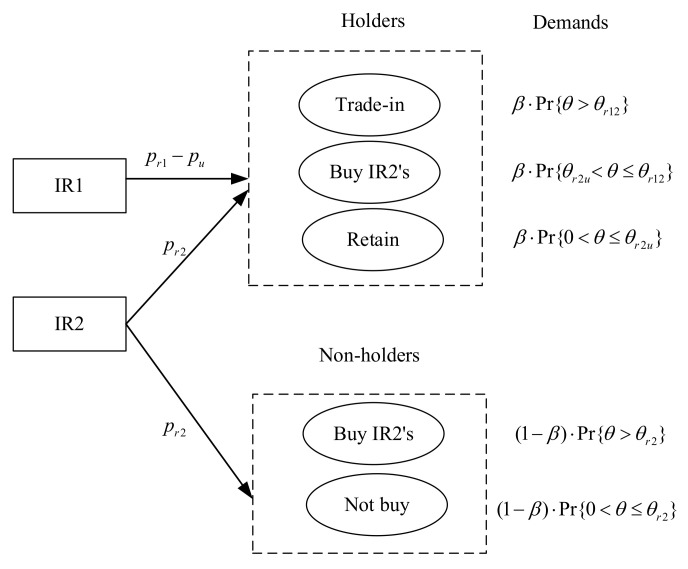
Consumer choices and demand for products from two IRs.

As with studies that focus on the competition between firms selling new and remanufactured products [5,27,39], we reserve the first case to focus on the competition between two IRs selling remanufactured products. Therefore, the demand for products from two IRs are $D_{r1}=\beta \cdot \left(1-\frac{p_{r1}-p_{u}-p_{r2}}{1-\gamma }\right)$ and $D_{r2}=\beta \cdot \left(\frac{p_{r1}-p_{u}-p_{r2}}{1-\gamma }-\frac{p_{r2}}{\gamma -\alpha }\right)+\left(1-\beta \right)\cdot \left(1-\frac{p_{r2}}{\gamma }\right)$. The trade-in strategy adopted by the IR1 serves to guarantee whole remanufactured from used products (i.e., $D_{r1}=Q_{u1}$), while the IR2 cannot guarantee it (i.e., $D_{r2}\leq Q_{u2}$), and $Q_{u}=Q_{u1}+Q_{u2}$. Figure 1 illustrates game sequence of IRs and consumers and Figure 2 shows consumer choices and demand for products from two IRs.

Given the above assumptions, the IR1’s profit and the constraint condition are

(1) $$\underset{p_{r1}}{max}\pi_{IR1}=\left(p_{r1}-p_{u}-c_{r}\right)\cdot D_{r1},$$

(2) $$s.t.0<D_{r1}\leq Q_{u},$$

where $D_{r1}$ constitutes not only the demand for products from the IR1, but also its collection quantities.

Similarly, the IR2’s profit and the constraint condition are

(3) $$\underset{p_{r2}}{max}\pi_{IR2}=\left(p_{r2}-c_{r}\right)\cdot D_{r2}-f\cdot \left(Q_{u}-D_{r1}\right),$$

(4) $$s.t.0<D_{r2}\leq Q_{u}-D_{r1},$$

where $D_{r2}$ constitutes the demand from the IR2, $Q_{u}-D_{r1}$ being the collection quantities by the IR2.

We have assumed that two IRs have the capacity to choose to implement a trade-in scenario or not (illustrated in Figure 2) and this affects consumer choices accordingly. We can determine the equilibrium decisions of profits by applying the Lagrangian and the Karush–Kuhn–Tucker (KKT) optimality conditions.

## 4. Equilibrium Analysis

As shown in Table 3, when ${\hat{p}}_{r2b}<p_{r2}<{\hat{p}}_{r2a}$ or ${\hat{p}}_{r2b}<{\hat{p}}_{r2a}<p_{r2}$, holders will not accept a trade-in strategy from the IR1 (i.e., $D_{r1}=0$). For the sake of tractability and given our focus on the competition between two IRs, this paper keeps the price low (i.e., $p_{r2}<{\hat{p}}_{r2b}<{\hat{p}}_{r2a}$) to facilitate sales of remanufactured products to consumers from both the IR1 and IR2. From Equations (1) and (3), and the constraint conditions (2) and (4), equilibrium decisions are as follows.

**Lemma** **1.** If $Q_{u}>{\hat{Q}}_{u}$, we have $p_{r1I}^{*}=\frac{1+c_{r}+2p_{u}-\gamma +p_{r2I}^{*}}{2}$ and $p_{r2I}^{*}=\frac{c_{r}\left[2\left(1-\alpha \right)\gamma -2\left(1-\beta \right)\left(\gamma^{2}+\alpha \right)-\beta \left(1-\gamma +c_{r}\right)\left(\gamma -\alpha \right)\gamma ]+[\beta f-\left(1-\beta \right)\left(1-\gamma \right)\right]2\left(\gamma -\alpha \right)\gamma }{\left[2\left(\beta +\alpha \beta -2\alpha \right)+c_{r}\beta \left(\gamma +\alpha -2\right)\right]\gamma }$; otherwise, we have $p_{r1II}^{*}=\frac{1+c_{r}+2p_{u}-\gamma +p_{r2II}^{*}}{2}$ and $p_{r2II}^{*}=\frac{\left(1-Q_{u}\right)\left(\gamma -\alpha \right)\gamma }{\gamma -\alpha +\alpha \beta }$. Further, we can obtain $D_{r1}^{*}$, $D_{r2}^{*}$, $\pi_{IR1}^{*}$, and $\pi_{IR2}^{*}$.

**Proof of all Lemmas and Propositions are given in the Appendix A, Appendix B, Appendix C, and Appendix D.**

Note that $Q_{u}>{\hat{Q}}_{u}$ represents an oversupply of used products (i.e., $Q_{u}-D_{r1}-D_{r2}>0$). In the alternate case, two IRs prefer to collect and remanufacture all used products (i.e., $Q_{u}-D_{r1}-D_{r2}=0$). Whether used products are oversupplied or not, there is a similar relationship between the prices of remanufactured products from the IRs (i.e., $p_{r1i}^{*}=\left(1+c_{r}+2p_{u}-\gamma +p_{r2i}^{*}\right)/2$, i=I,II). This is because $D_{r1}$ constitutes both the demand for the IR1 products and its collection quantities. Whereas for the IR2, the demand $D_{r2}$ and the collection quantities $Q_{u}-D_{r1}$ are inconsistent if $Q_{u}>{\hat{Q}}_{u}$. Consequently, the IR1 determines the price of remanufactured products to be more stable and subject to the effects of $p_{r2}$.

Under the trade-in scenario, we find that $p_{u}$ has not only influenced the consumer utilities and demand ($U_{r1}$ and $D_{r1}$), but also influenced the equilibrium price $p_{r1}^{*}$. Without the trade-in strategy, f is unchanged with respect to consumer utilities, demand, and equilibrium price. Although both $p_{u}$ and f are exogenous and given, the impact of the trade-in strategy on consumers and IRs is significant. In addition, the impact of the proportion of holders β on prices is as follows.

**Proposition** **1.** In the case of an oversupply of used products, if $c_{r}<{\hat{c}}_{r}$, we have $\partial p_{r1I}^{*}/\partial \beta <0$ and $\partial p_{r2I}^{*}/\partial \beta <0$; otherwise, $\partial p_{r1I}^{*}/\partial \beta \geq 0$ and $\partial p_{r2I}^{*}/\partial \beta \geq 0$. In the alternate case, we have $\partial p_{r1II}^{*}/\partial \beta <0$ and $\partial p_{r2II}^{*}/\partial \beta <0$.

Proposition 1 implies that, in the case of an oversupply of used products with a low unit production cost of remanufactured products (i.e., $Q_{u}>{\hat{Q}}_{u}$ and $c_{r}<{\hat{c}}_{r}$) or when all used products are collected and remanufactured (i.e., $Q_{u}\leq {\hat{Q}}_{u}$), the prices are decreased with the proportion of holders (i.e., $\partial p_{r1}^{*}/\partial \beta <0$ and $\partial p_{r2}^{*}/\partial \beta <0$). Under this situation, the IR2 can select either an improved quality performance or adopt remanufacturing practices that create less damage to the environment, which is more attractive to non-holders. Therefore, the IR2 can expect to collect and produce more remanufactured products. The more holders that exist in the market, the less incentive the IR2 has to remanufacture and sell remanufactured products. However, our findings provide a counter-intuitive result for the IR1, since it gains a larger consumer market as the proportion of holders increases. We contend that, for some holders, there would be a preference to buy remanufactured products from the IR2 or retain their used products as opposed to engaging in a trade-in scheme. The IR1 is compelled to decrease the price of its products in order to attract more holders to engage in its trade-in strategy and buy its remanufactured goods.

If all the consumers are holders (i.e., β=1), the equilibrium decisions are as follows:

**Proposition** **2.** Assume at first β=1. We have $p_{r1}^{*}=\frac{1+c_{r}+2p_{u}-\gamma +p_{r2}^{*}}{2}$ and $p_{r2}^{*}=\frac{\left(1+2f-\gamma \right)\left(\gamma -\alpha \right)+c_{r}\left(2-3\alpha +\gamma \right)}{4-3\alpha -\gamma }$. Further, we can obtain $D_{r1}^{*}$, $D_{r2}^{*}$, $\pi_{IR1}^{*}$, and $\pi_{IR2}^{*}$. In addition, we have $D_{r1}^{*}>D_{r2}^{*}$ and $\pi_{IR1}^{*}>\pi_{IR2}^{*}$.

Proposition 2 reveals that if all the consumers are holders, the IR1 is more benefit than the IR2 no matter for the demand or profit (i.e., $D_{r1}^{*}>D_{r2}^{*}$ and $\pi_{IR1}^{*}>\pi_{IR2}^{*}$). As we have analyzed in Proposition 1, although we find the impact of the proportion of holders on price is negative, a high proportion of holders (e.g., β=1) is beneficial to demand levels and profit for the IR1.

## 5. Numerical Experiments

**Figure 3:**
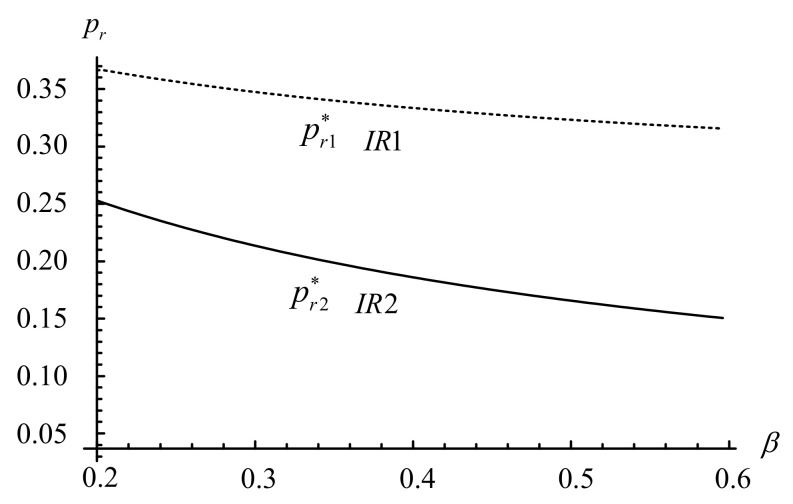
Impact of β on the IR’s prices. Notes: * means the optimal solution in Figure 3, Figure 4, Figure 5, Figure 6.

**Figure 4:**
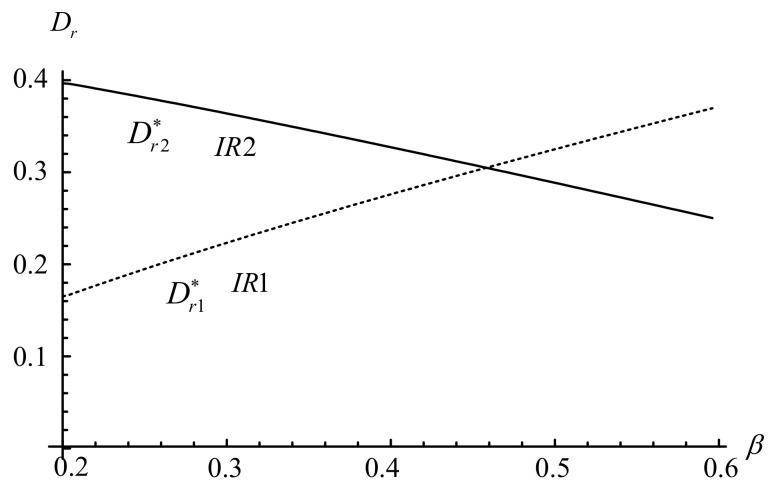
Impact of β on the IR’s demands.

**Figure 5:**
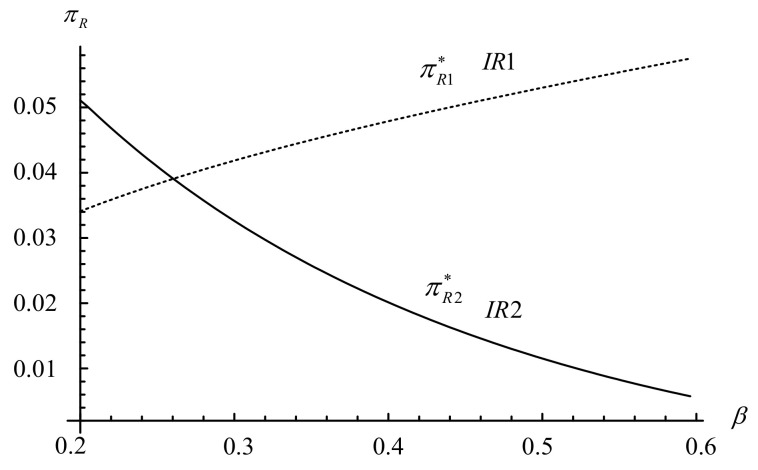
Impact of β on the IR’s profits.

**Figure 6:**
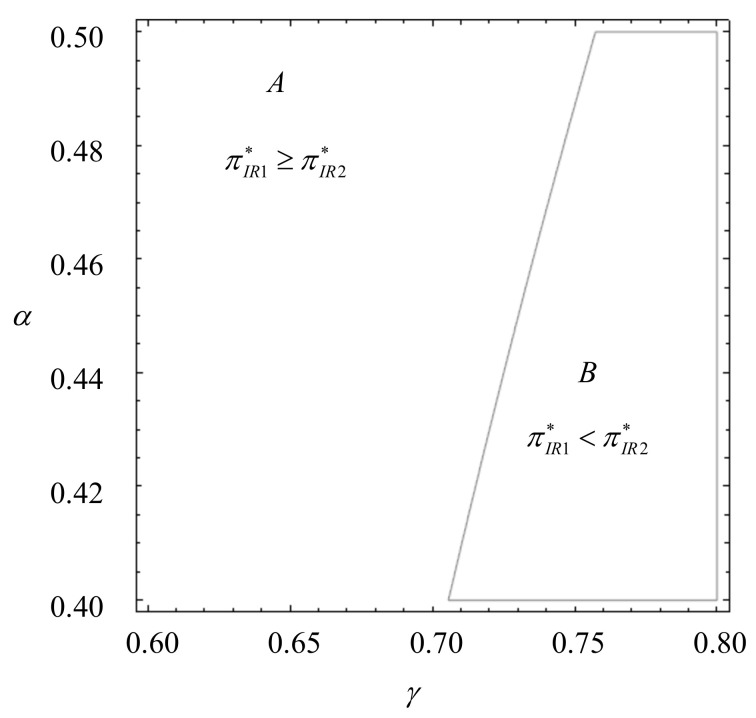
Impact of α and γ on the IR’s profits.

This section conducts several numerical tests to complement the aforementioned analysis and to illustrate how key factors affect the IRs’ prices, demands, and profits. The default values are as follows: β=0.25, f=0.02, $c_{r}=0.09$, $p_{u}=0.07$, $Q_{u}=0.84$, α=0.45, and γ=0.75. These values ensure that the assumptions of 0<α<γ<1, $f<p_{u}$, and $0\leq \beta ,Q_{u}\leq 1$ are satisfied in the experiment. Figure 3, Figure 4, Figure 5, Figure 6 illustrate the impacts of α, γ, β and on the IR’s decisions and profits.

Figure 3 and Figure 4 illustrate that the prices of remanufactured products from the IR1 and IR2 decrease as the proportion of holders reduces (i.e., $\partial p_{r1}^{*}/\partial \beta <0$ and $\partial p_{r2}^{*}/\partial \beta <0$). However, when the proportion of holders increases, the IR1’s demand increases whereas the IR2’s demand decreases (i.e., $\partial D_{r1}^{*}/\partial \beta >0$ and $\partial D_{r2}^{*}/\partial \beta <0$). By lowering the price of remanufactured products, the IR1 attracts more holders to participate in its trade-in strategy, generating more demand for its remanufactured products. However, for the IR2, as the unit collection fee is far less than the price of used products under the trade-in strategy (i.e., $f<p_{u}$), the demand still decreases even with a reduction in prices of remanufactured products.

From Figure 5, we find that the impact of the proportion of holders on the IR’s profits are similar to that of the impact of the proportion of holders on the IR’s demands (i.e., $\partial \pi_{R1}^{*}/\partial \beta >0$ and $\partial \pi_{R2}^{*}/\partial \beta <0$). When the proportion of holders is sufficiently low (i.e., β≤0.26), the appeal of a trade-in strategy is limited, which does not improve the IR1’s profit (i.e., $\pi_{R1}^{*}<\pi_{R2}^{*}$). However, when the proportion of holders is high (i.e., β>0.26), the IR1’s profit is greatly increased by the growth in demand and exceeds that of the IR2’s profit (i.e., $\pi_{R1}^{*}\geq \pi_{R2}^{*}$). That is, the trade-in strategy creates a win-win situation for consumers (especially for holders) and for the IR1 (resulting in “small profits but quick turnover”).

As shown in Figure 6, the IR1’s profit in region A (i.e., γ−α>0.31 and some special parameters in 0.26≤γ−α≤0.31) is higher than that of the IR2’s (i.e., $\pi_{R1}^{*}\geq \pi_{R2}^{*}$), in that the trade-in strategy brings higher revenues. This is because when consumers experience a greater difference in durability between the remanufactured products and original new products, trade-in strategies are more welcomed by consumers. Conversely, if consumers choose to purchase the IR2’s remanufactured products instead of participating in the IR1’s trade-in strategy, the trade-in strategy brings lower revenues (i.e., if γ−α<0.26, then $\pi_{R1}^{*}<\pi_{R2}^{*}$).

## 6. Conclusions

As public concerns about sustainability and the conservation of natural resources continue to grow, consumers are starting to look towards the use of remanufactured goods as an alternative and viable solution. Trade-in strategies enable independent manufacturers to participate in the circular economy, while other manufacturers choose to simply sell remanufactured products without trade-in schemes. With this in mind, we developed game-theoretic models to identify the conditions in which trade-in schemes are beneficial to independent manufacturers. Our results can be applied to CLSCs wherein independent manufacturers either adopt trade-in strategies or not.

When used products are in oversupply and there is a low unit production cost of remanufactured products, or when all used products are collected and remanufactured, the price of remanufactured goods falls as the proportion of holders decreases. However, when the proportion of holders increases, demand for the IR1 products, obtained through a trade-in strategy, increases, whereas demand for the IR2 products decreases. The greater the proportion of holders, the greater the competitive advantage the IR1 gains over the IR2. We also that when consumers experience a greater difference in durability between remanufactured products and new products, there will be a greater uptake of trade-in strategies which brings high revenues to the IR1.

### 6.1. Theoretical Implications

This paper contributes to the theory about trade-in strategy by: (i) investigating trade-in strategy in the presence of holders who own used products and non-holders who do not own used products; and (ii) examining the impact of the proportion of the holders, the willingness-to-pay for remanufactured products, and durability of new products on the decisions and trade-in strategy choice. Unlike the extant literature [7,14,27], we study the competition between remanufactured products and focus on the roles of characteristics of consumer behavior in making decisions. Considering consumer factors [4], we clearly describe the consumers’ heterogeneity preference for different remanufactured products when the IR provides trade-in strategy.

### 6.2. Managerial Implications

Our results have managerial implications for independent manufacturers who are deciding whether a trade-in strategy, in the presence of competition, is advantageous. Our results reveal that prices, demand, and profits are affected by the proportion of holders, consumer’s WTP discount rate for remanufactured products, and the durability of new products. In particular, we offer the following insights:

- When there are all holders exist in the market, independent manufacturers who adopt trade-in strategy obtain more benefit than others no matter for the demand or profit;
- The higher the proportion of holders in the market, the lower the prices of remanufactured goods, irrespective of whether independent manufacturers offer trade-in schemes or not;
- Independent manufacturers driven by self-serving goals will be inclined to adopt a “trade-old-for remanufactured” strategy when the proportion of holders is sufficiently high;
- If remanufactured goods are very popular with holders and original new products are not durable, independent manufacturers who are seeking maximum benefits should adopt a trade-in strategy.

### 6.3. Future Research

A number of potential avenues for future research stem from our work. An investigation into how the trade-in strategy changes with government interventions (e.g., government subsidy or carbon emissions tax) for remanufacturing operations would be instructive. Secondly, our model has only focused on the “trade-old-for-remanufactured” framework, whereas future researchers could consider “trade-old-for-new” models and compare these two trade-in strategies in CLSC operations. Finally, more attention could be paid to behavioral factors (e.g., strategic behavior and environmentally responsible behavior) in the future.

## Figures and Tables

**Table 1:** Comparison of this study and related literature.

Studies	Consumer Perspective	Trade-in	Competition	Main Features of the Paper
Savaskan et al. [11]	Not considered	Not considered	Not considered	Three options for remanufacturing
Ferrer and Swaminathan [26]	Consumer’s WTP	Not considered	New vs. Re	Two-period remanufacturing
Atasu et al. [4]	Consumer’s WTP and green segment	Not considered	New vs. Re	Existence of a green consumer segment
Choi et al. [14]	Consumer’s WTP	Not considered	New vs. Re	Channel leadership and coordination in CLSC
Örsdemir et al. [27]	Consumer’s WTP	Not considered	New vs. Re	Competitive in quality of products
Agrawal et al. [7]	Consumer’s WTP	TON	New vs. New	Impact of trade-in rebates on price discrimination
Ma et al. [33]	Consumer’s WTP	TON and TOR	New vs. Re	Comparison between two kinds of trade-ins
Xiao [8]	Consumer’s WTP	TON	New vs. Re	Exchange decisions and sharing mechanism
Zhang and Zhang [9]	Consumer’s WTP	TON	New vs. Re	Impact of strategic behavior on trade-in remanufacturing
Feng et al. [35]	Consumer’s WTP	TON	New vs. Re	Trade-in program in secondary market
Chen et al. [36]	Consumer’s WTP	TON	New vs. Re	Implement trade-in programs and advertising decisions in CLSC
Kleber et al. [30]	Consumer’s WTP	TON and TOR	New vs. Newand Re vs. Re	Competition between two remanufacturers
This study	Consumer’s WTP and holder segment	TOR	Re vs. Re	Trade-in between two IRs in presence of competition

WTP: Willingness-to-pay; TON: Trade-old-for-new; TOR: Trade-old-for-remanufactured; Re: Remanufactured.

**Table 2:** The parameters and variables used in our model.

Symbol	Definition
**Model parameters**	
θ	Consumer’s willingness-to-pay (WTP) for remanufactured products from the IR1,$\;\theta \sim U\;\left[0,1\right]$
γ	Consumer’s WTP a discount rate for remanufactured products from the IR2, γ<1
β	Proportion of holders, 0≤β≤1
α	Durability of new products, 0<α<γ
$c_{r}$	Unit production cost of remanufactured products
$Q_{u}$	Total number of used products for acquisition, $0\leq Q_{u}\leq 1$
$Q_{u1}$	Collection quantities by the IR1, $Q_{u1}=D_{r1}$
$Q_{u2}$	Collection quantities by the IR2, $Q_{u2}=Q_{u}-Q_{u1}$
$p_{u}$	Price of used products under trade-in strategy by the IR1
f	Unit collection fee of used products by the IR2, $f<p_{u}$
**Functions**	
$U_{r1}$,$D_{r1}$	Utility and demand of remanufactured products from the IR1
$U_{r2}$,$D_{r2}$	Utility and demand of remanufactured products from the IR2
$U_{u}$	Utility of used products
**Decision variables**	
$p_{r1}$	Price of remanufactured products by the IR1
$p_{r2}$	Price of remanufactured products by the IR2

**Table 3:** Demand for remanufactured products in different contexts.

	Demand	$D_{r1}$	$D_{r2}$
Cases			
$p_{r2}<{\hat{p}}_{r2b}<{\hat{p}}_{r2a}$		$\beta \cdot \mathrm{Pr}\{\theta >\theta_{r12}\}$	$\beta \cdot \mathrm{Pr}\{\theta_{r2u}<\theta \leq \theta_{r12}\}+\left(1-\beta \right)\cdot \mathrm{Pr}\{\theta >\theta_{r2}\}$
otherwise		0	$\beta \cdot \mathrm{Pr}\{\theta >\theta_{r2u}\}+\left(1-\beta \right)\cdot \mathrm{Pr}\{\theta >\theta_{r2}\}$

Notes: ${\hat{p}}_{r2a}=\frac{\left(1-\alpha \right)\left(p_{r1}-p_{u}\right)}{2-\alpha -\gamma }$ and ${\hat{p}}_{r2b}=\frac{\left(\gamma -\alpha \right)\left(p_{r1}-p_{u}\right)}{1-\alpha }$.

## Data Availability

Data is contained within the article.

## Appendix A. Proof of the Demand

Solving $\theta_{r12}=\theta_{r1u}$ yields $p_{r2}={\hat{p}}_{r2a}=\frac{\left(1-\alpha \right)\left(p_{r1}-p_{u}\right)}{2-\alpha -\gamma }$. Further, if $p_{r2}<{\hat{p}}_{r2a}$, $\theta_{r12}>\theta_{r1u}$; otherwise, $\theta_{r12}\leq \theta_{r1u}$. Solving $\theta_{r12}=\theta_{r2u}$ yields $p_{r2}={\hat{p}}_{r2b}=\frac{\left(\gamma -\alpha \right)\left(p_{r1}-p_{u}\right)}{1-\alpha }$. Further, if $p_{r2}<{\hat{p}}_{r2b}$, $\theta_{r12}>\theta_{r2u}$; otherwise, $\theta_{r12}\leq \theta_{r2u}$. Solving $\theta_{r12}=\theta_{r2u}$ yields $p_{r2}={\hat{p}}_{r2c}={\hat{p}}_{r2b}$. Further, if $p_{r2}<{\hat{p}}_{r2b}$, $\theta_{r1u}>\theta_{r2u}$; otherwise, $\theta_{r1u}\leq \theta_{r2u}$. From $p_{u}<p_{r1}$ and 0<α,γ<1, we obtain ${\hat{p}}_{r2a}-{\hat{p}}_{r2b}=\frac{\left(p_{r1}-p_{u}\right){\left(1-\gamma \right)}^{2}}{\left(2-\alpha -\gamma \right)\left(1-\alpha \right)}>0$. Thus, we consider three cases: ①$p_{r2}<{\hat{p}}_{r2b}<{\hat{p}}_{r2a}$, i.e., $\theta_{r12}>\theta_{r1u}>\theta_{r2u}$; ②${\hat{p}}_{r2b}<p_{r2}<{\hat{p}}_{r2a}$, i.e., $\theta_{r2u}>\theta_{r12}>\theta_{r1u}$; ③${\hat{p}}_{r2b}<{\hat{p}}_{r2a}<p_{r2}$, i.e., $\theta_{r2u}>\theta_{r1u}>\theta_{r12}$. Further, we can obtain the demands in three cases. In addition, the demands in case ② and ③ are identical.

## Appendix B. Proof of Lemma 1

Considering constraint conditions of $0<D_{r1}\leq Q_{u}$ and $0<D_{r2}\leq Q_{u}-D_{r1}$, the Lagrangian and the KKT optimality conditions for two IRs optimal problem are

(A1) $$L_{IR1}\left(p_{r1},\mu \right)=\left(p_{r1}-p_{u}-c_{r}\right)\cdot D_{r1}+\mu \left(Q_{u}-D_{r1}\right),$$

(A2) $$L_{IR2}\left(p_{r2},\lambda \right)=\left(p_{r2}-c_{r}\right)\cdot D_{r2}-f\cdot \left(Q_{u}-D_{r1}\right)+\lambda \left(Q_{u}-D_{r1}-D_{r2}\right),$$

(A3) $$\alpha L_{IR1}/\alpha p_{r1}=\frac{\beta \left(1+c_{r}-2p_{r1}+p_{r2}+2p_{u}-\gamma +\mu \right)}{1-\gamma }=0,$$

(A4) $$\alpha L_{IR2}/\alpha p_{r2}=c_{r}\cdot D_{r2}+\frac{\left(p_{r2}-c_{r}-\lambda \right)\cdot \left[\left(1-\alpha \right)\gamma -\left(1-\beta \right)\left(\gamma^{2}+\alpha \right)\right]}{\left(1-\gamma \right)\left(\gamma -\alpha \right)\gamma }-\frac{\beta \left(f-\lambda \right)}{1-\gamma }=0,$$

(A5) $$\mu \left(Q_{u}-D_{r1}\right)=0,$$

(A6) $$\lambda \left(Q_{u}-D_{r1}-D_{r2}\right)=0,$$

$0<D_{r1}\leq Q_{u}$, $0<D_{r2}\leq Q_{u}-D_{r1}$, μ≥0, λ≥0.

Given the Lagrangean multipliers μ and λ, there are four possible cases. The first case (denoted by I) implies that $\mu^{*}=0$ and $\lambda^{*}=0$, which is equal to $Q_{u}-D_{r1}>0$ and $Q_{u}-D_{r1}-D_{r2}>0$. From 0<β,γ<1, the second-order condition of $\pi_{IR1}$ over $p_{r1}$ is $\frac{\partial^{2}\pi_{IR1}}{\partial p_{r1}^{2}}=-\frac{2\beta }{1-\gamma }<0$, i.e., the second-order condition is satisfied. Similarly, from 0<α<γ<1, the second-order condition of $\pi_{IR2}$ over $p_{r2}$ is $\frac{\partial^{2}\pi_{IR2}}{\partial p_{r2}^{2}}=-2\left\{\frac{\left(\gamma -\alpha \right)\left(1-\gamma \right)+\beta \left[\gamma^{2}-\alpha \left(2\gamma -1\right)\right]}{\left(\gamma -\alpha \right)\left(1-\gamma \right)\gamma }\right\}<0$, which is satisfied. Solving the conditions of $\alpha L_{IR1}/\alpha p_{r1}=0$ and $\alpha L_{IR2}/\alpha p_{r2}=0$ for $\left(p_{r1},p_{r2}\right)$, we have $p_{r1I}^{*}$ and $p_{r2I}^{*}$. Inserting $p_{r1I}^{*}$ and $p_{r2I}^{*}$ into $Q_{u}-D_{r1}-D_{r2}=0$, generates ${\hat{Q}}_{u}$. Further, if $Q_{u}>{\hat{Q}}_{u}=1-\frac{\left[\beta \gamma -\left(1-\beta \right)\left(\gamma -\alpha \right)\right]p_{r2I}^{*}}{\left(\gamma -\alpha \right)\gamma }$, then $Q_{u}-D_{r1}-D_{r2}>0$.

The second case (denoted by II) implies that $\mu^{*}=0$ and $\lambda^{*}>0$, which is equal to $Q_{u}-D_{r1}>0$ and $Q_{u}-D_{r1}-D_{r2}=0$. From $\frac{\partial^{2}\pi_{IR1}}{\partial p_{r1}^{2}}=-\frac{2\beta }{1-\gamma }<0$ and $Q_{u}-D_{r1}-D_{r2}=0$, we solve the conditions of $\alpha L_{IR1}/\alpha p_{r1}=0$ and $Q_{u}-D_{r1}-D_{r2}=0$ for $\left(p_{r1},p_{r2}\right)$. Then we have $p_{r1II}^{*}$ and $p_{r2II}^{*}$.

The third case (denoted by III) implies that $\mu^{*}>0$ and $\lambda^{*}=0$, which is equal to $Q_{u}-D_{r1}=0$ and $Q_{u}-D_{r1}-D_{r2}>0$, which does not exist.

The fourth case (denoted by IV) implies that $\mu^{*}>0$ and $\lambda^{*}>0$, which is equal to $Q_{u}-D_{r1}=0$ and $Q_{u}-D_{r1}-D_{r2}=0$, which means $D_{r2}=0$, i.e., there is no point in studying the competition.

## Appendix C. Proof of Proposition 1

From Lemma 1, we solve $\partial p_{r2I}^{*}/\partial \beta =0$ for $c_{r}$ and obtain ${\hat{c}}_{r}=\min\{\frac{4}{3}\left[\frac{\alpha \left(1-\gamma \right)}{\gamma -\alpha }-f\right]+\frac{2+\gamma }{3},\frac{2+\gamma -f}{3}\}$. Further, if $c_{r}<{\hat{c}}_{r}$, then $\partial p_{r2I}^{*}/\partial \beta <0$, $\frac{\partial p_{r1I}^{*}}{\partial \beta }=\frac{1}{2}\frac{\partial p_{r2I}^{*}}{\partial \beta }<0$; otherwise, $\partial p_{r1I}^{*}/\partial \beta \geq 0$ and $\partial p_{r2I}^{*}/\partial \beta \geq 0$. Similarly, we can obtain $\frac{\partial p_{r1II}^{*}}{\partial \beta }=\frac{1}{2}\frac{\partial p_{r2II}^{*}}{\partial \beta }=-\frac{\alpha \left(1-Q_{u}\right)\left(\gamma -\alpha \right)\gamma }{2{\left(\gamma -\alpha +\alpha \beta \right)}^{2}}<0$.

## Appendix D. Proof of Proposition 2

Similar to Lemma 1, we omit the proof of the equilibrium prices. From $p_{r1}^{*}$, $p_{r2}^{*}$ and 0<α<γ<1 $D_{r1}^{*}-D_{r2}^{*}=\frac{\left(1-\gamma +2f\right)\left(\gamma -\alpha \right)\left(1-\alpha \right)+c_{r}\left(1-\gamma \right)\left(2-\alpha -\gamma \right)}{\left(\gamma -\alpha \right)\left(1-\gamma \right)\left(4-3\alpha -\gamma \right)}>0$, $\pi_{IR1}^{*}-\pi_{IR2}^{*}=\left(p_{r1}^{*}-p_{u}-c_{r}\right)\cdot D_{r1}^{*}-\left(p_{r2}^{*}-c_{r}\right)\cdot D_{r2}^{*}+f\cdot \left(Q_{u}-D_{r1}^{*}\right)>\left(p_{r1}^{*}-p_{u}-c_{r}\right)\cdot D_{r1}^{*}-\left(p_{r2}^{*}-c_{r}\right)\cdot D_{r2}^{*}+f\cdot D_{r2}^{*}=\left(p_{r1}^{*}-p_{u}-c_{r}\right)\cdot D_{r1}^{*}-\left(p_{r2}^{*}-c_{r}-f\right)\cdot D_{r2}^{*}$. From 0<α<γ<1, we have $\left(p_{r1}^{*}-p_{u}-c_{r}\right)-\left(p_{r2}^{*}-c_{r}-f\right)=\frac{\left(1-\gamma +2f\right)\left(2-\alpha -\gamma \right)+c_{r}\left(1-\gamma \right)}{4-3\alpha -\gamma }>0$ and $D_{r1}^{*}-D_{r2}^{*}>0$. Therefore, we have $\pi_{IR1}^{*}>\pi_{IR2}^{*}$.
